# Diet Shapes Mortality Response to Trauma in Old Tephritid Fruit Flies

**DOI:** 10.1371/journal.pone.0158468

**Published:** 2016-07-06

**Authors:** James R Carey, Pablo Liedo, Cong Xu, Jane-Ling Wang, Hans-Georg Müller, Yu-Ru Su, James W Vaupel

**Affiliations:** 1 Department of Entomology, University of California Davis, Davis, California, United States of America; 2 Center for the Economics and Demography of Aging, University of California, Berkeley, California, United States of America; 3 El Colegio de la Frontera Sur (ECOSUR), Tapachula, Chiapas, Mexico; 4 Department of Radiology, School of Medicine, Stanford University, Stanford, California, United States of America; 5 Department of Statistics, University of California Davis, Davis, California, United States of America; 6 Fred Hutchinson Cancer Research Center, University of Washington, Seattle, Washington, United States of America; 7 Max Planck Institute for Demographic Research, Konrad-Zuse-Strasse 1, Rostock, Germany; Leibniz Institute on aging—Fritz Lipmann Institute (FLI), GERMANY

## Abstract

Despite the importance of trauma in healthspan and lifespan in humans as well as in non-human species, with one important exception the literature in both gerontology and ecology contains virtually no experimental demographic studies concerned with trauma in any species. We used dietary manipulation [full diet (F) versus sugar-only (S)] to produce four levels of frailty in 55-day old tephritid fruit flies (*Anastrepha ludens*) that were then subject to the trauma of cage transfer stress (n = 900/sex in each of the 4 treatments). The key results included the following: (1) there is a trauma effect caused by the transfer that depends on previous diet before transfer, new diet after transfer and gender of the fly; (2) males are more vulnerable than females; (3) if initial diet was F, flies are relatively immune against the trauma, and the subsequent diet (F or S) does not matter; (4) however if initial diet was S, then the effect of the trauma depends largely on the diet after the transfer; (5) flies transferred from S to F diets do very well in terms of remaining longevity (i.e. greatest remaining longevity), while flies transferred from S to S diet do poorly (i.e. shortest remaining longevity). We discuss both the strengths and weaknesses of this study and implications of the results.

## Introduction

Despite the importance of accidents and trauma among the elderly [1] and the increased use of model organisms for studies on healthspan [2,3] including studies on the effects on longevity of injury and stress on aging in fruit flies [4–6], it is remarkable that so few studies concerned with healthspan have been conducted on trauma in these [7] or any other invertebrates. Because of this dearth of investigations on trauma on model organisms in general and insects in particular, we initiated an investigation using the tephritid fruit fly, *Anastrepha ludens* (Mexican fruit fly), to determine how different frailty levels affect the mortality response of older flies. The study used dietary manipulations to produce two cohorts of flies that differed in frailty which were then subjected to a trauma caused by transfer to a different environment. We recorded daily survival during the entire post-transfer period but divided the analysis into two parts including short term survival (i.e. the post-transfer survival response during the first six days to quantify the severity of the acute trauma) and long term survival (including all remaining lifetimes after transfer). We used these results to address the following sets of questions: (1) How does trauma shape gender-specific mortality in older flies of different frailty levels and what role does diet play? (2) Is trauma-induced short-term (acute) mortality either magnified or diminished for older frail flies? (3) Are the long-term consequences of different frailty levels permanent or temporary?

## Materials and Methods

### Basic design

All flies for experiments were obtained as pupae from the Moscafrut rearing facility in Tapachula, Mexico and, as adults maintained at 25±2 C and 65±5% RH and with access to food and water. To produce older flies with different levels of frailty for the experiments approximately 2,500 newly-eclosed adult flies of both sexes were placed in each of 60 mesh-covered aluminum cages measuring 15 X 60 X 90-cm and with full diet (3:1 ratio of sugar-to-yeast hydrolysate i.e. protein source) in half of the cages and sugar-only in the other half. At 55 days, an age when approximately 10 to 20% (depending on diet) of the flies remained alive, technicians used a mouth aspirator to capture and then transfer individual flies from these large group cages to much smaller individual plexiglass 4 X 4 X 10-cm cages where their survival and female reproduction was monitored daily through death. Half of the flies were fed the same diets both pre- and post-transfer and the other half were fed the alternative diet (all food and water replenished daily). The design concept thus yielded four treatments based on pre- and post-transfer dietary combinations i.e. sugar-to-sugar (SS), sugar-to-full (SF), full-to-sugar (FS), and full-to-full (FF). Full diet consisted of a mixture of 3 parts sugar to 1 part yeast hydrolysate.

An average of 900 individuals of each were transferred in each of the four treatments. Logistical demands preempted the use of pre-transfer control cohorts. With from 10 to 20% survival to age 55, to ensure a pool of >10,000 55-day old live flies would have required 50,000 to 100,000 newly-eclosed flies caged individually and their survival monitored and their husbandry needs (i.e. food; water) met daily throughout the pre-transfer period. Although our research resources (i.e. technical help; space; time) were adequate for generating a large data set measuring post-transfer mortality, they were insufficient for additionally housing and monitor this large numbers of flies pre-transfer.

### Trauma sources

The trauma to which all older (55 day-old) flies were subjected included the traumatic sum total resulting from three components of the transfer process from group to individual cages: (1) *Extraction impact* (i.e. impact in aspiration tube when removed); (2) *Transfer impact* (i.e. impact when gently blown into the new cage); and (3) *Husbandry stress* (i.e. placement into a new environment).

### Statistical methods for trauma—short- and long-term effects

Models were fitted with the R package. To quantify the short-term effects of trauma for male and female flies under the various diet combinations, we applied logistic regression analysis to compare the probability of death within 1–6 days after transfer (i.e., the probability of a fly living for less than 6 days after transfer) and that of death after 6 days (i.e., the probability of a fly living for more than 6 days after transfer). The response in this model is the indicator whether the death occurred within 1–6 days or in more than 6 days after transfer (1: death in 1–6 days, 0: death after 6 days) and the three predictors are gender (0:female, 1:male); diet1 (diet before transfer, sugar only: 1, full: 0; diet2 (diet after transfer, same coding as for diet1). Defining $\tilde{p}$ as the probability of a fly dying in the first six days after transfer, we fitted models with the predictors and their interactions and omitting non-significant terms led to the model with two interaction terms (where we included all main effects contributing to an interaction term if the interaction term was included)

$$logit\left(\tilde{p}\right)=\alpha_{0}+\alpha_{1}gender+\alpha_{2}diet_{1}+\alpha_{3}diet_{2}+\alpha_{4}gender\;\times diet_{2}+\alpha_{5}diet_{1}\times diet_{2}$$ (1)

To study the long term effects of trauma on mortality for males and for females under the various treatment combinations, denoting T = remaining lifetime after transfer, i.e. age at death for each fly minus age at transfer, we fitted a linear regression of log(T) on the predictors, which were the same as described above. The response is the total lifetime after transfer, *T*, and again there are three binary covariates as in the logistic regression: “gender” (1:male, 0:female), “diet1” (diet before transfer, 1: diet with sugar only, 0: diet with protein) and “diet2” (diet after transfer, same coding as for diet1). Applying the log-transformation to the response and the final model including significant covariates is:

$$\text{log}\left(T\right)=\gamma_{0}+\gamma_{1}gender+\gamma_{2}diet_{1}+\gamma_{3}diet_{2}+e,\;\cdots$$ (2)

where $e∼N\left(0,\sigma_{0}^{2}\right)$. We note that all lifetimes were fully observed and no censoring occurred. Nevertheless, an alternative to Model (2) is a Cox proportional hazards regression model. We implemented a Cox regression model and obtained the same qualitative results as described here.

## Results

This study involved a total of 7,200 individual flies subjected to the same set of traumas at the same age. The differences among the 900 individuals in each of the treatment groups were due to either a fly’s gender or to 1-of-4 dietary combinations of early (0 to 55 days) or late (>55 days) sugar-only or full diet. The large number of individuals used in each treatment was required to assure that, despite high initial mortality in some treatments (e.g. male sugar-only), the numbers of individuals remaining alive post-transfer would permit statistically-meaningful analyses of longer term effects on survival. This need for large initial numbers of flies of both sexes in four treatments at advanced age (55 days) preempted logistically the inclusion of control cohorts where individual flies are maintained in solitary confinement throughout their lives (i.e. not subject to transfer trauma).

**Fig 1:**
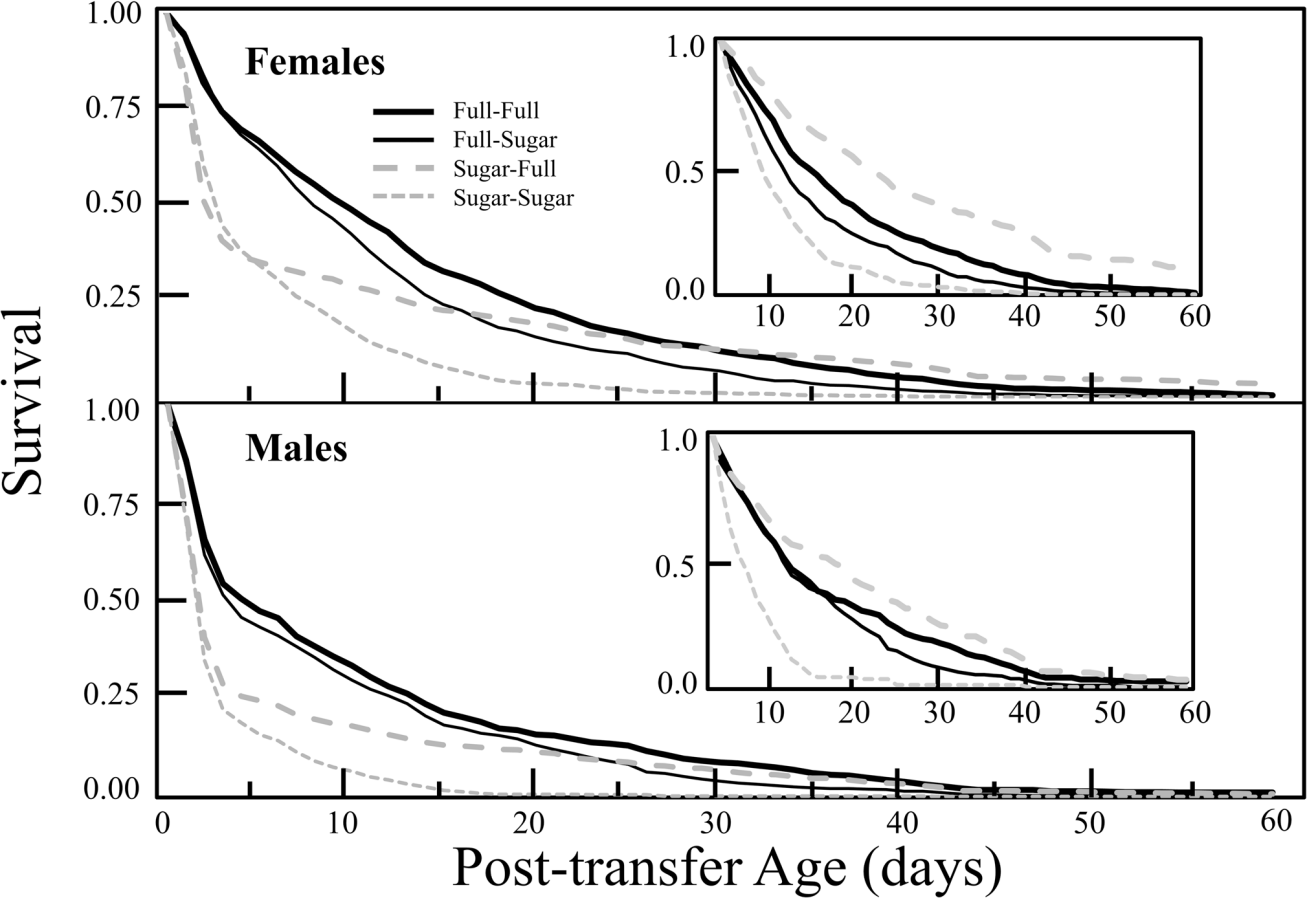
Fruit fly gender-specific post-transfer remaining survival function estimates for each of four dietary switch treatments, separately for male and female flies. Insets depict additional remaining survival function estimates for remaining survival 6 days after transfer to assess the longer term survival prospects for those flies which survive the first 6 days of post-transfer stress.

**Fig 2:**
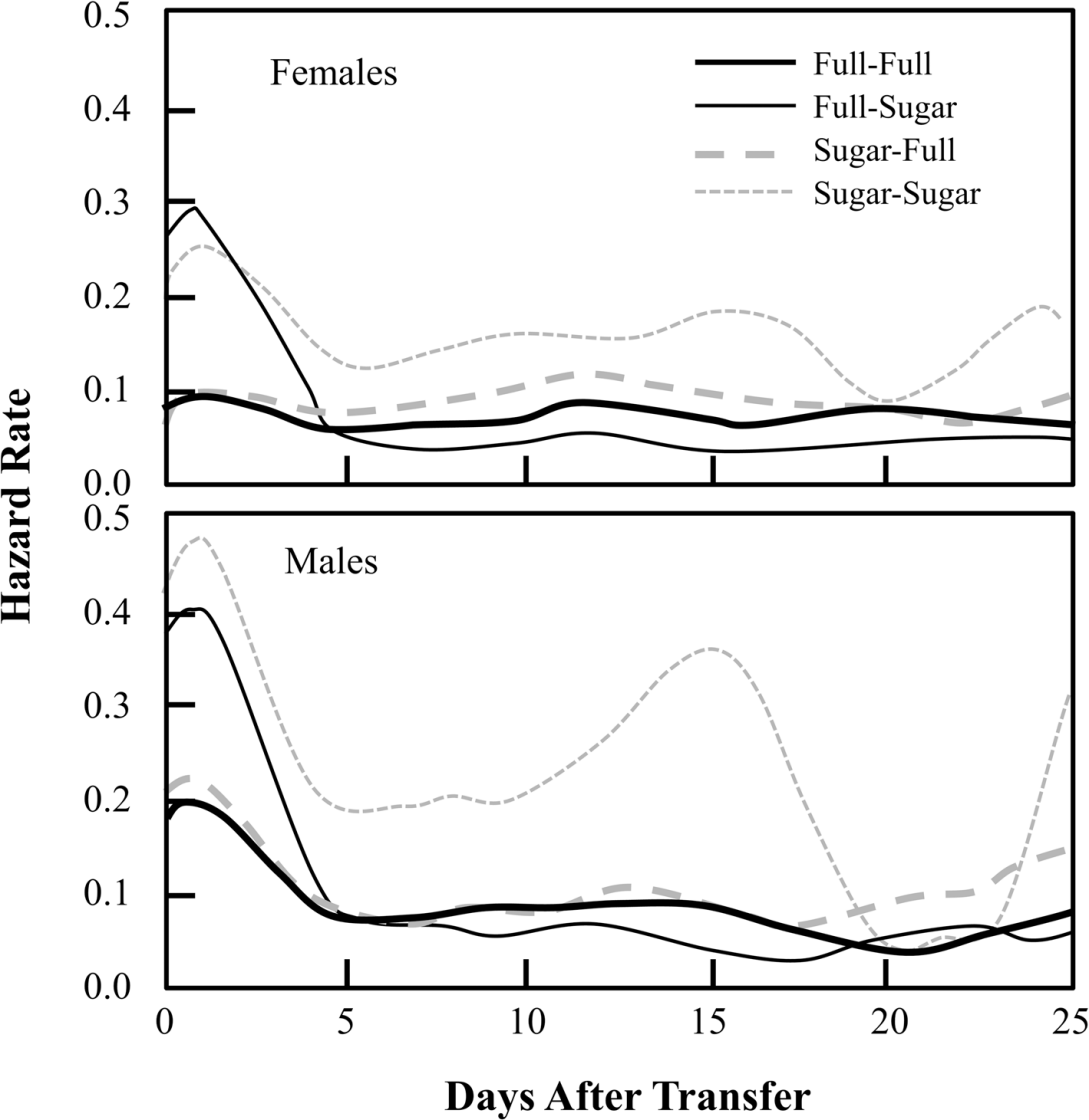
Post-transfer age-specific hazard rates for each of four treatments, separately for males and females. The hazard rate (or force of mortality) quantifies the instantaneous risk of death at a specific time, given that a subject is still alive and is at risk. Depicted are hazard rates for females (top) and for males (bottom) for four different diet groups, characterized by four pre- and post-transfer diet combinations.

The post-transfer survival function (Fig 1) and hazard rate (Fig 2) patterns for all treatments and both male and female flies merit several comments. First, survival rates for both male and female flies in all treatments decreased rapidly (and hazard rates spiked) during the first few days after transfer indicating that all flies experienced significant short-term (acute) trauma. Second, the magnitudes of the mortality surges (Fig 2 and Table 1) when quantified as probability of death within the first 6 days after transfer were consistently higher for males, for the cohorts which were maintained on sugar-only pre-transfer diets, and for flies that were switched from sugar to full diet. For example, depending on treatment the probability of dying within the first 6 days after transfer ranged from 0.38 to 0.70 for females and from 0.54 to 0.85 for males.

**Table 1:** Fitted probabilities and odds of death by sex and diet pattern within six days post-transfer, obtained from fitting the model in Eq (1). The “Odds Ratio” column reported in the table refers to the odds ratios of each specific diet pattern described in the first column vs. the full-full diet within each gender strata. The odds listed in the table provide a measure of the severity of the immediate trauma, but not of long-term mortality (CIs are 95% confidence intervals).

Combination	Prob. of death within 1–6 days	Odds of death within 1–6 days	Odds Ratio	95% CI for Odds Ratio
**Females**				
Full-Full	0.382	0.619	--	(0.550, 0.696)
Full-Sugar	0.388	0.634	1.025	(0.866, 1.212)
Sugar-Full	0.652	1.876	3.033	(2.637, 3.489)
Sugar-Sugar	0.702	2.357	3.810	(3.207, 4.527)
**Males**				
Full-Full	0.536	1.153	--	(1.027, 1.295)
Full-Sugar	0.600	1.499	1.300	(1.101, 1.535)
Sugar-Full	0.778	3.499	3.033	(2.637, 3.489)
Sugar-Sugar	0.848	5.575	4.833	(4.017, 5.816)

Third, whereas the flies in the sugar-to-full treatment experienced the highest probability of death during the first 6 days, the survivors in this treatment experienced the highest long-term remaining survival after age 65. This switch is evident in Fig 1 insets showing that overall survival in flies experiencing sugar-to-full diets is lowest relative to all other treatments when considered from the first day post-transfer. However, when one targets instead remaining survival for flies in all treatments at day 6 (Fig 1 insets) survival is the highest in the sugar-to-full treatment for both males and females. The cross-over in mortality driving the overall survival patterns is reflected in Fig 2 where, for both males and females, flies in the sugar-to-full diet experienced high mortality immediately after transfer but low mortality after 5 to 7 days post-transfer.

We find that all parameters in the Model 1 are positive and highly significant (Table 2), except for diet 2 after transfer, pointing to the presence of short-term trauma effects on subsequent mortality. Specifically, we have the following findings: (i) as ${\hat{\alpha }}_{1}>0$, the short term trauma effect is worse for males compared to females; (ii) as ${\hat{\alpha }}_{2}>0$, flies starting on sugar diet experience more trauma effect; (iii) as ${\hat{\alpha }}_{4}>0$, sugar diet after trauma is additionally detrimental for males regardless of the pre-trauma diet, while this effect is not present for females. So full diet after trauma protects males but does not protect females; and (iv) as ${\hat{\alpha }}_{5}>0$, flies that have been raised on sugar and continue on sugar after trauma experience more trauma effect than those that had protein either before and/or after trauma. This is independent of gender.

**Table 2:** Parameters and their estimates for the odds that death occurs within the first 6 days after trauma, rather than later, as obtained from a logistic regression model, quantifying the impact of trauma on the initial post-traumatic mortality. Here *α*_0_ is the intercept, *α*_1_ the parameter for sex (0 = female, 1 = male), *α*_2_ the parameter for pre-transfer diet (0 = full, 1 = sugar only), *α*_3_ the parameter for post-transfer diet, *α*_4_ the parameter for the interaction of sex and post-transfer diet, and *α*_5_ the parameter for the interaction of pre- and post-transfer diet (where post-transfer diet is coded in the same way as pre-transfer diet). The model is given in Eq (1).

Parameter	Estimate	Standard Error	P-value^a^
*α*_0_	-0.480	0.060	1.48 × 10^−15^ ***
*α*_1_	0.623	0.071	< 2 × 10^−16^ ***
*α*_2_	1.110	0.071	< 2 × 10^−16^ ***
*α*_3_	0.024	0.086	0.777
*α*_4_	0.238	0.103	0.021 *
*α*_5_	0.204	0.104	0.050 *

^**a**^ * and *** indicate that p-value is smaller or equal to 0.05 and 0.001, respectively.

So in terms of optimal treatment after trauma (i.e. highest survival), the following complex picture emerges: For males it is always better to have access to protein after trauma, irrespective of what they had access to before. For females, if they were raised on protein the after diet does not matter much. But if they were raised on sugar, protein is better. Looking at the regression coefficients of the effects, protein before transfer is the best protection against trauma effect, being female is the second best, then for males having protein after trauma is next, and the smallest but still significant effect is having protein either before or after and this latter effect applies to both males and females (since ${\hat{\alpha }}_{2}>{\hat{\alpha }}_{1}>{\hat{\alpha }}_{4}>{\hat{\alpha }}_{5}>0$). Thus resilience to trauma is increased by protein and being female. The effect of trauma is mitigated by giving protein to females which had access to sugar-only diet pre-trauma and to males irrespective of what they had access to during the earlier pre-trauma period.

The results for the analyses of overall life expectancy from Model 2 (see Tables 3 and 4) demonstrate that flies under diet FF (full diet pre- and post-transfer) have the longest expected total lifetime after transfer followed by diet FS (full-to-sugar), SF (sugar-to-full) and SS (sugar-to-sugar) in a descending order. Female flies have longer expected total lifetime after transfer than male flies under the same diet sequence. These conclusions are consistent with our observations from Fig 1 and also with the logistic regression analysis on the short term trauma effects. Comparing the three effects with negative regression coefficients: Worst trauma effect on longevity is associated with sugar before transfer, second worst with being male, and third worst with sugar diet after transfer (since ${\hat{\gamma }}_{2}<{\hat{\gamma }}_{1}<{\hat{\gamma }}_{3}<0$). This means protein after transfer cannot make up for the damaging effect of a sugar diet before transfer or for being male.

**Table 3:** Parameters and their estimates for model (2), regressing log(T) on various predictors, where T is the remaining lifetime of a fly after transfer. The predictors are indicators coding gender, pre-transfer and post-transfer diet, where the coding of these indicators is as in the legend of Table 2, and *σ*_0_ is the standard deviation of the error.

Parameter	Estimate	Standard Error	t-value	P-value
*γ*_0_	2.146	0.026	81.18	< 2 × 10^−16^
*γ*_1_	-0.461	0.026	-17.43	< 2 × 10^−16^
*γ*_2_	-0.661	0.026	-25.01	< 2 × 10^−16^
*γ*_3_	-0.177	0.026	-6.70	2.25 × 10^−11^
*σ*_0_	1.122	NA	NA	NA

**Table 4:** Expected remaining lifetimes for the various cohorts (FPP = females with protein before and after transfer) after transfer, obtained from the fitted model (2) with parameter estimated given in Table 3; the expected remaining lifetimes are all significantly different as evidenced by the p-values in Table 3.

	Combination	Expected Total Lifetime *E*(*T*)	Estimated Value
Females	FF	$\text{exp}\;\left(\gamma_{0}+\frac{\sigma_{0}^{2}}{2}\right)$	16.04
	FS	$\text{exp}\;\left(\gamma_{0}+\gamma_{3}+\frac{\sigma_{0}^{2}}{2}\right)$	13.44
	SF	$\text{exp}\;\left(\gamma_{0}+\gamma_{2}+\frac{\sigma_{0}^{2}}{2}\right)$	8.28
	SS	$\text{exp}\;\left(\gamma_{0}+\gamma_{2}+\gamma_{3}+\frac{\sigma_{0}^{2}}{2}\right)$	6.94
Males	FF	$\text{exp}\;\left(\gamma_{0}+\gamma_{1}+\frac{\sigma_{0}^{2}}{2}\right)$	10.12
	FS	$\text{exp}\;\left(\gamma_{0}+\gamma_{1}+\gamma_{3}+\frac{\sigma_{0}^{2}}{2}\right)$	8.48
	SF	$\text{exp}\;\left(\gamma_{0}+\gamma_{1}+\gamma_{2}+\frac{\sigma_{0}^{2}}{2}\right)$	5.22
	SS	$\text{exp}\;\left(\gamma_{0}+\gamma_{1}+\gamma_{2}+\gamma_{3}+\frac{\sigma_{0}^{2}}{2}\right)$	4.38

## Discussion

Although a large literature exists on the effects on mortality in fruit flies of various types of stressors such as desiccation and starvation [4,8] and of mechanical impairments [9], ours is only the second controlled study we are aware of that documents the immediate consequences of acute trauma [7] in any organism and the only study which did so for the effects of trauma on the remaining life expectancy. Our experimental approach enabled us to analyze the: (i) actuarial manifestations of old age frailty independent of the effects of behavioral selection (i.e. frailty as underlying propensity *for* an accident versus source of vulnerability *to* an accident); (ii) mortality differences in trauma when the intensity and types of trauma are standardized across all treatments; and (iii) effects on both acute and remaining life expectancy of pre-traumatic environmental (nutritional) conditions.

For logistical reasons (see Methods) cohorts of flies for all treatments that were not subjected to transfer could not be included, and thus the absolute size of the traumatic effect of the transfer on survival in comparison to undisturbed flies could not be quantified. However, our comparisons of the effects of diets before and after the transfer show that the trauma effects are modulated by diet, and their severity in terms of shortened survival depends on the combination of pre- and post-transfer diets.

Our study has several important strengths. The first involves the use of formal statistical criteria to both define and determine the existence of acute trauma. We fit a logistic regression model to determine the odds that death occurs within the first 6 days after trauma, rather than later. A second strength was the use of very large initial numbers of older flies (i.e. 55 days). These large numbers increase the statistical power when testing for the effects of diet on the actuarial outcome of both trauma (i.e. short term mortality) and life expectancies at older ages, given previous and current diets. A third strength was our study’s focus on the effects of trauma in both sexes and at older ages. Our results underscore the complexity of mortality assessment in general and (for the current case) at older ages in particular. Although the effect on fruit fly mortality of switching diets is well documented [10,11], comparative studies involving either both males and females or the effects of trauma are absent from the current literature.

Our study also has a weakness—the lack of non-traumatized flies as controls for determining the absolute effects of trauma in both the short and long term. As noted earlier, logistical demands preempted this possibility because, depending on initial diet, only from 10 to 20% of the initial flies were still alive at the time of transfer (i.e. transfer at 55 days) from the group cages to the individual ones. Thus to include baseline control cohorts of non-traumatized individuals would have required that the initial number of flies to be monitored in individual cages for nearly two months would have been 50,000 to 100,000 flies pre-transfer—numbers that exceeded our logistical capabilities. We thus opted for a design that provided insights into relative rather than absolute effects of early diet mortality trauma.

Our results have at least three implications. The first is technical—transfer of insects in experiments is a source of trauma that has the potential to have significant, if not catastrophic, effects on both short- and long-term mortality. This can potentially have major consequences on the outcome of experiments, particularly those in which mortality is one of the key experimental metrics. The second implication relates to the incidence of traumatic events in natural populations. Inasmuch as violence is a frequent occurrence in many species (e.g. male-male competition) a more systematic approach to understanding the short- and long-term effects of trauma will add depth, scope and nuance to future studies on aging in the wild [12]. The third implication is that, because the effects on mortality of trauma in humans in particular and in vertebrate animals in general cannot be studied systematically (i.e. baseline controls; standardized conditions), trauma studies would forever remain an epidemiological and descriptive science rather than experimental or clinical one without the use of invertebrates. Thus the only organisms that can be used in experimental studies in biogerontology concerning the effects of trauma on healthspan and aging will be those involving fruit flies [2] or other invertebrates.

## Supporting Information

S1 Table Original data for Mexfly males. Results of trauma studies for male Mexflies subjected to each of four treatments.
(XLSX)

S2 Table Original data for Mexfly females. Results of trauma studies for female Mexflies subjected to each of four treatments.
(XLSX)
